# The effect of travel restrictions on the spread of the 2019 novel coronavirus (COVID-19) outbreak

**DOI:** 10.1126/science.aba9757

**Published:** 2020-03-06

**Authors:** Matteo Chinazzi, Jessica T. Davis, Marco Ajelli, Corrado Gioannini, Maria Litvinova, Stefano Merler, Ana Pastore y Piontti, Kunpeng Mu, Luca Rossi, Kaiyuan Sun, Cécile Viboud, Xinyue Xiong, Hongjie Yu, M. Elizabeth Halloran, Ira M. Longini, Alessandro Vespignani

**Affiliations:** 1 Laboratory for the Modeling of Biological and Socio-technical Systems, Northeastern University, Boston, MA, USA.; 2 Bruno Kessler Foundation, Trento, Italy.; 3 ISI Foundation, Turin, Italy.; 4 Fogarty International Center, NIH, Bethesda, MD, USA.; 5 School of Public Health, Fudan University, Key Laboratory of Public Health Safety, Ministry of Education, Shanghai, China.; 6 Fred Hutchinson Cancer Research Center, Seattle, WA, USA.; 7 Department of Biostatistics, University of Washington, Seattle, WA, USA.; 8 Department of Biostatistics, College of Public Health and Health Professions, University of Florida, Gainesville, FL, USA.

## Abstract

Motivated by the rapid spread of coronavirus disease 2019 (COVID-19) in mainland China, we use a global metapopulation disease transmission model to project the impact of travel limitations on the national and international spread of the epidemic. The model is calibrated on the basis of internationally reported cases and shows that, at the start of the travel ban from Wuhan on 23 January 2020, most Chinese cities had already received many infected travelers. The travel quarantine of Wuhan delayed the overall epidemic progression by only 3 to 5 days in mainland China but had a more marked effect on the international scale, where case importations were reduced by nearly 80% until mid-February. Modeling results also indicate that sustained 90% travel restrictions to and from mainland China only modestly affect the epidemic trajectory unless combined with a 50% or higher reduction of transmission in the community.

## Outbreak to pandemic

In response to global dispersion of severe acute respiratory syndrome–coronavirus 2 (SARS-CoV-2), quarantine measures have been implemented around the world. To understand how travel and quarantine influence the dynamics of the spread of this novel human virus, Chinazzi *et al.* applied a global metapopulation disease transmission model to epidemiological data from China. They concluded that the travel quarantine introduced in Wuhan on 23 January 2020 only delayed epidemic progression by 3 to 5 days within China, but international travel restrictions did help to slow spread elsewhere in the world until mid-February. Their results suggest that early detection, hand washing, self-isolation, and household quarantine will likely be more effective than travel restrictions at mitigating this pandemic.

## Teaser

A SARS-CoV-2 epidemiological model reveals the effects of travel restrictions and transmission reduction efforts on the spread of this novel virus.

Beginning in December 2019, Chinese health authorities have been closely monitoring a cluster of pneumonia cases in the city of Wuhan in Hubei province, China. The pathogen that causes the viral pneumonia in affected individuals is the newly recognized coronavirus known as severe acute respiratory syndrome–coronavirus 2 (SARS-CoV-2) (*1*). As of 3 March 2020, 80,151 cases (*2*) have been detected and confirmed in mainland China. Internationally, more than 10,566 additional cases have been detected and confirmed in 72 countries (*3*). In this work, we model both the domestic and international spread of the novel coronavirus disease 2019 (COVID-19) epidemic. We estimate the effects of the travel ban implemented in Wuhan and the international travel restrictions adopted by several countries in early February 2020.

To model the international spread of the COVID-19 outbreak, we used the global epidemic and mobility model (GLEAM), an individual-based, stochastic, and spatial epidemic model (*4*–*7*). GLEAM uses a metapopulation network approach integrated with real-world data where the world is divided into subpopulations centered around major transportation hubs (usually airports). The subpopulations are connected by the flux of individuals traveling daily among them. The model includes more than 3200 subpopulations in roughly 200 different countries and territories. The airline transportation data encompass daily origin-destination traffic flows from the Official Aviation Guide (OAG) and International Air Transport Association (IATA) databases (updated in 2019), whereas ground mobility flows are derived from the analysis and modeling of data collected from the statistics offices of 30 countries on five continents (*5*). Mobility variations in mainland China were derived from Baidu location-based services (LBS). Within each subpopulation, the human-to-human transmission of COVID-19 is modeled by using a compartmental representation of the disease in which individuals can occupy one of the following states: susceptible, latent, infectious, and removed. Susceptible individuals can acquire the virus through contacts with individuals in the infectious category and can subsequently become latent (i.e., infected but not yet able to transmit the infection). Latent individuals progress to the infectious stage at a rate inversely proportional to the latent period (which we assume to have the same duration as the incubation period), and infectious individuals progress to the removed stage at a rate inversely proportional to the infectious period. The sum of the mean latent and infectious periods defines the generation time. Removed individuals are those who can no longer infect others (i.e., they are isolated, hospitalized, have recovered, or have died).

The model generates an ensemble of possible epidemic scenarios described by the number of newly generated infections, time of disease arrival in each subpopulation, and number of traveling infection carriers. We assume a starting date of the epidemic that falls between 15 November 2019 and 1 December 2019, with 40 infections caused by zoonotic exposure (*8*–*11*). The transmission dynamic is calibrated by using an approximate Bayesian computation approach (*12*) to estimate the posterior distribution of the basic reproductive number *R*_0_ by exploring the likelihood of importation of COVID-19 infections to international locations (*13*). We assume that the overall global detection of imported infections can be as low as 40% (*14*, *15*). Data on importation of cases were obtained from available published line lists (*16*, *17*).

We performed a sensitivity analysis by considering different combinations of average latent and infectious periods, detection rates, initial conditions, and a generation time (*T*_g_) ranging from 6 to 11 days on the basis of plausible ranges from the SARS epidemic and recent analysis of COVID-19 data (*16*, *18*–*23*). Details and sensitivity analysis on all parameters are reported in the supplementary materials (*12*). Here we report the results for *T*_g_ = 7.5 days (*20*). The obtained posterior distribution provides an average *R*_0_ = 2.57 [90% confidence interval (CI): 2.37 to 2.78] and a doubling time of *T*_d_ = 4.2 days (90% CI: 3.8 to 4.7 days). The obtained values are in the same range as previous analyses based on early COVID-19 data (*9*, *20*, *24*–*26*). Although the calibration obtained for different generation times provides different posterior distributions for *R*_0_, in the early stages of the epidemic the prevalence of infections and case importations is determined by the epidemic growth rate, and the obtained results (*12*) are consistent with those reported here.

## Wuhan travel ban

On 22 January 2020, the projected median number of infections with no travel restrictions for mainland China, excluding Wuhan, was 7474 (90% CI: 3529 to 16,142). The overwhelming majority of infections were in Wuhan with a median number of 117,584 (90% CI: 62,468 to 199,581). To analyze the effect of the travel ban from Wuhan, we implemented long-range travel restrictions beginning on 23 January (airport shutdown). Furthermore, we modeled mobility limitations within mainland China by using de-identified and aggregated domestic population movement data between Chinese provinces for February 2020, as derived from Baidu LBS (*12*).

**Fig. 1:**
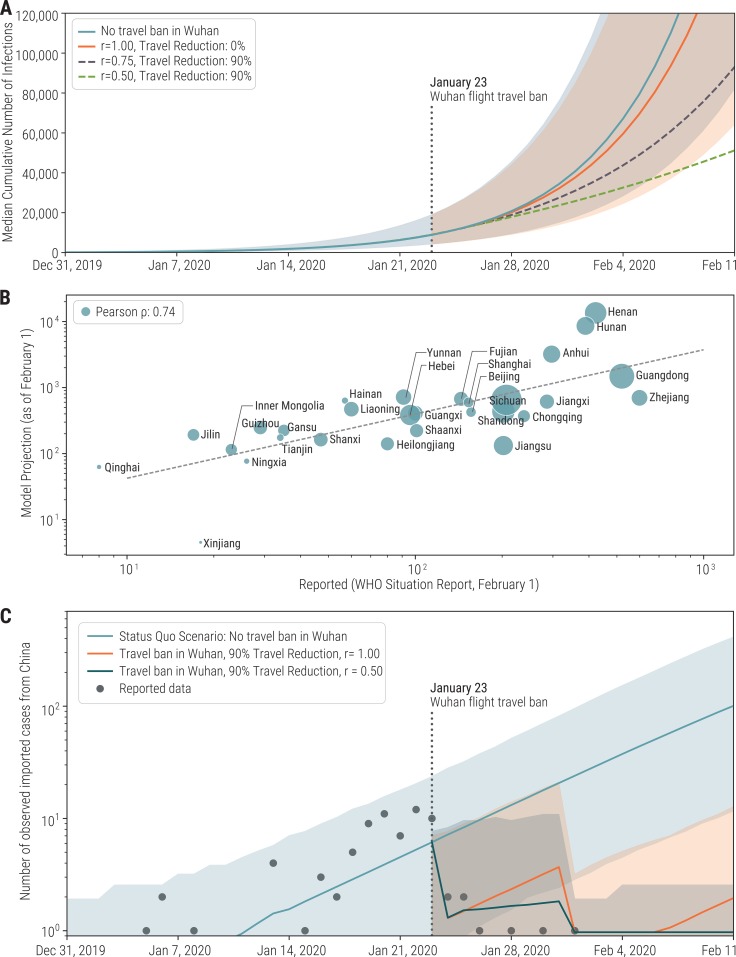
Effect of the Wuhan travel ban on the COVID-19 epidemic. (**A**) Trajectory of the COVID-19 epidemic in Chinese locations (excluding Wuhan) under the ban on travel to and from Wuhan as of 23 January 2020. Trajectories are also plotted for scenarios with relative transmissibility reduction *r* and international travel restrictions. Lines represent median cumulative number of infections; shaded areas represent 90% reference ranges. (**B**) Correlation between the number of cases reported in each province by the WHO situation report and model projections on 1 February 2020 (no provinces were reporting zero cases by this date). Circle size is proportional to the population size in each province. (**C**) Projections of the average detected number of daily international case importations for different modeling scenarios. Shaded areas represent 99% reference ranges. We report the observed data of international case importations with a travel history from China, classified by arrival date. We also report scenarios with relative transmissibility reduction *r*. Data points after 23 January 2020 were used for out-of-sample validation and were not used in the model calibration.

**Fig. 2:**
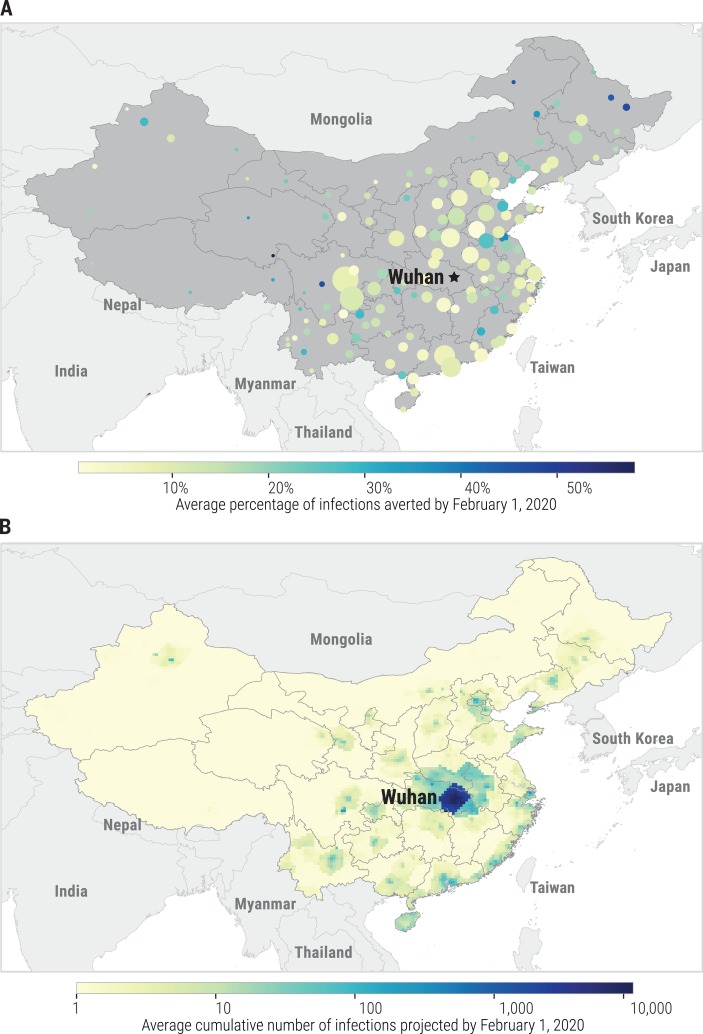
Effects of Wuhan travel ban on COVID-19 incidence across mainland China. (**A**) Relative incidence reduction as of 1 February 2020. Circle color represents the relative reduction in the number of infections, whereas circle size corresponds to population. (**B**) Projected cumulative number of infections by the same date, after implementation of travel restrictions in Wuhan. A resolution of 0.25° by 0.25° geographical cells was used in the model.

Initially, we assumed no changes in transmissibility and disease dynamics (the status quo scenario). The model output shows no noticeable differences in the epidemic trajectory of Wuhan but a delay of ~3 days for other locations in mainland China (Fig. 1A). The overall reduction of infections in mainland China, excluding Wuhan, was close to 10% by 31 January 2020, with a relative reduction of infections across specific locations ranging from 1 to 58% (Fig. 2). With a doubling time of 4 to 5 days, this level of reduction corresponds to only a modest delay (1 to 6 days) of the epidemic trajectory in mainland China. These results are in agreement with estimates from the combination of epidemiological and human mobility data (*27*). The model clearly indicates that, as of 23 January 2020, the epidemic was seeded in several locations across mainland China. As an independent validation test, we assessed the cumulative number of cases in mainland China provinces through 1 February 2020 (Fig. 1B), as reported from the official World Health Organization (WHO) situation report (*28*), and compared these results with model projections. The model projections are highly correlated with the observed data (Pearson’s correlation coefficient = 0.74, *P* < 0.00001), although, as expected, we found that there are significantly fewer reported cases than projected (Fig. 1B). If we assume that the number of reported cases in the WHO situation report and in the simulation are related through a simple binomial stochastic sampling process, we find that the median ascertainment rate of detecting an infected individual in mainland China is 24.4% (interquartile range: 12.7 to 35.8%). In other words, the modeling results suggest that, in mainland China, only one in four infections is detected and confirmed.

## Relative risk of case importation

**Fig. 3:**
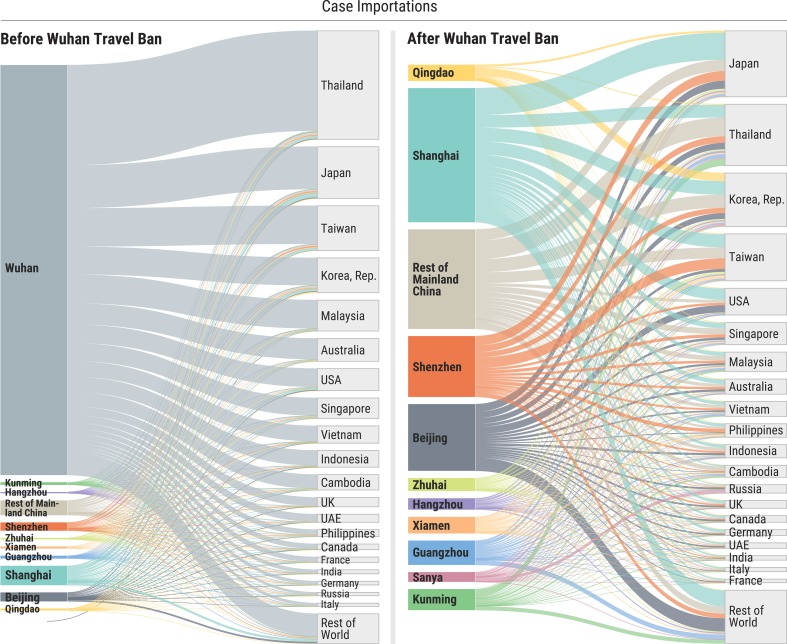
Relative risk of case importation. Contribution to the relative risk of importation from the 10 Chinese cities with the highest rates of disease (plus the rest of mainland China) until 22 January 2020 (**left**) and after the Wuhan travel ban from 23 January to 1 March 2020 (**right**). The listed countries are the 20 countries at greatest risk of case importation. Flows are proportional to the relative probability that a single imported case will travel from a given origin to a specific destination.

The model also allows us to estimate the number of case importations in international locations from mainland China. In Fig. 1C, we report the mean number of total international importation events in a fully status quo scenario as opposed to a travel ban. We find a 77% reduction in cases imported from mainland China to other countries as a result of the Wuhan travel ban in early February. Although the number of cases imported internationally decreases markedly at first, it picks up again in the following weeks with importation from locations in mainland China. The model indicates that, after the travel restrictions in Wuhan are implemented on 23 January, the five origin cities with the highest rates of international case importations are Shanghai, Beijing, Shenzhen, Guangzhou, and Kunming. Similarly, the model can rank countries across the world according to the relative risk of importing cases from mainland China. More precisely, the relative risk is defined for each country *Y* as the relative probability *P*(*Y*) that a single infected individual travels from an area affected by the epidemic to that specific destination *Y*. In other words, given the occurrence of one exported case, *P*(*Y*) is the relative probability that the disease carrier will appear in location *Y*, with respect to any other possible location. This risk depends on the travel flow from cities in mainland China to other countries and the disease prevalence in those cities. Notably, the traffic flows used in the model are origin-destination data that do not depend on traveling routes (i.e., a proxy for the actual mobility demand across cities). Figure 3 illustrates how the cities with the most COVID-19 cases in mainland China contribute to the relative risk of the 20 countries that are most susceptible to case importation, both before and after implementation of the Wuhan travel ban . In particular, before the travel ban, ≈86% of the internationally imported cases originated from Wuhan. After the travel ban, the top 10 contributors to the relative risk—of which the top three are Shanghai (28.1%), Beijing (14%), and Shenzhen (12.8%)—accounted for at least ≈80% of the internationally imported cases. The countries most at risk of importation after the implementation of the Wuhan travel ban are Japan (11% pre-ban, 13.9% post-ban), Thailand (22.8% pre-ban, 13% post-ban), the Republic of Korea (7.4% pre-ban, 11.3% post-ban), Taiwan (9.5% pre-ban, 10% post-ban), and the United States (4.7% pre-ban, 5.7% post-ban).

## International travel restrictions and transmissibility reduction

**Fig. 4:**
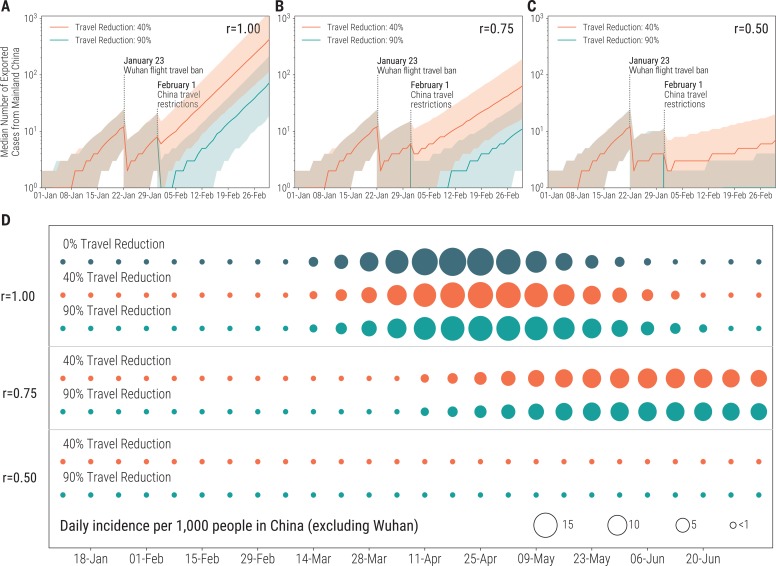
Combined effects of travel and transmissibility reductions on the epidemic. (**A**) Median total number of imported infections from mainland China with no transmissibility reduction and travel reductions of 40 and 90%. (**B**) Same as (A) for the moderate transmissibility reduction scenario (*r* = 0.75). (**C**) Same as (A) for the strong transmissibility reduction scenario (*r* = 0.5). Shaded areas represent 90% CIs. (**D**) Disease incidence in mainland China, excluding Wuhan, for the scenarios plotted in (A) to (C).

Starting in early February 2020, 59 airline companies suspended or limited flights to mainland China, and several countries—including the United States, Russia, Australia, and Italy—have imposed government-issued travel restrictions (*29*–*34*). It is difficult to calculate exactly the level of traffic reduction imposed by these measures. For this reason, we analyzed two major scenarios in which international travel restrictions produce a 40 and 90% overall traffic reduction to and from mainland China. A relative reduction of transmissibility could be achieved through early detection and isolation of cases, as well as behavioral changes and awareness of the disease in the population. Along with travel reductions, we considered three scenarios pertaining to disease transmissibility: (i) a status quo situation with the same transmissibility as that from the model calibration through 23 January 2020; (ii) a moderate relative reduction of the original transmissibility (25%), corresponding to a transmissibility dampening factor of *r* = 0.75; and (iii) a strong reduction (50%) of the original transmissibility (*r* = 0.50). In Fig. 4, we show the combined effects of the travel and transmissibility reductions on the epidemic incidence in mainland China and the number of exported cases to other countries.

The simulated scenarios reveal that even in the case of 90% travel reductions (Fig. 4D), if transmissibility is not reduced (*r* = 1), the epidemic in mainland China would be delayed for no more than 2 weeks. The model projects that, in the status quo scenario, the peak of the epidemic in mainland China will be reached in late April to early May 2020. Notably, in the absence of transmissibility reductions, the epidemic would peak in Wuhan during the first week of March. The number of infections imported in other countries (Fig. 4, A to C) was initially affected by a 10-fold reduction, but by 1 March, when there is no transmissibility reduction (*r* = 1), we would again see 170 and 35 detected cases per day for the 40 and 90% travel restrictions scenarios, respectively. However, the concurrent presence of both travel and transmissibility reductions produces a much larger synergistic effect that becomes visible by delaying both the epidemic activity in mainland China and the number of internationally imported infections. In the moderate transmissibility reduction scenarios (*r* = 0.75), the epidemic peak is delayed to late June 2020, and the total number of international infection importations by 1 March is 26 cases per day for the 40% scenario and 5 per day for the 90% scenario. Even more restrictive travel limitations (>90%) would extend the period during which the importation of infections is greatly reduced. Strong transmissibility reduction (*r* = 0.5) along with travel restrictions would delay the epidemic growth in mainland China such that the daily incidence rate would never surpass 1 infection per 1000 people and the number of imported infections at international destinations would always be in the single-digit range. The effect of transmissibility reduction on the short-term epidemic curve in mainland China is also visible (Fig. 1A): There is a pronounced reduction in the number of infections by 22 February 2020, with respect to the status quo epidemic curve. We also report the estimated number of detected international importations, as determined by the model in the strong transmissibility reduction scenario (Fig. 1C). The results are consistent with the data collected from the travel history of international imported cases after 23 January 2020 (*16*, *17*). Similar results are obtained by assuming that the transmissibility reduction interventions successfully reduce the reproductive number below the epidemic threshold in the second half of February, as data from mainland China seem to suggest (*28*).

Notably, many infected individuals from mainland China have not been detected and have potentially dispersed to international locations. By 1 February 2020, in the strong transmissibility reduction scenario, the model estimates 101 (90% CI: 50 to 173) importation events, with one or more potential infections that could seed multiple epidemic outbreaks across the world, potentially leading to the international expansion of the COVID-19 epidemic. This finding is consistent with the emergence of COVID-19 outbreaks in countries such as Italy, the Republic of Korea, and Iran in the second half of February 2020.

Our analysis, as with all modeling exercises, has several limitations and requires certain assumptions. The model parameters, such as generation time and incubation period, are chosen on the basis of early data associated with the COVID-19 outbreak and prior knowledge of SARS and Middle East respiratory syndrome (MERS) coronavirus epidemiology. Although the model is stable to variations in these parameters, more information on the key characteristic of the disease would considerably reduce uncertainties. At this stage, the transmission and mobility model does not account for heterogeneities due to age differences in susceptibility and contact patterns. The model calibration does not consider correlations among importations (family travel) and assumes that travel probabilities are homogeneous across all individuals in the catchment area of each transportation hub. We were not able to find reliable data on the effectiveness of containment measures (e.g., body temperature screening for passengers on flights departing from Wuhan International Airport) in mainland China before 23 January, so this information is not included in the model. In the travel restriction scenario, we assume long-term enforcement of individual mobility restrictions (travel was restricted until the end of June 2020), but this policy may not be feasible or sustainable for such a long period.

## Discussion

The analysis of the COVID-19 outbreak and the modeling assessment of the effects of travel limitations could be beneficial to national and international agencies for public health response planning. We show that, by 23 January 2020, the epidemic had already spread to other cities within mainland China. The travel quarantine around Wuhan has only modestly delayed the spread of disease to other areas of mainland China. This finding is consistent with the results of separate studies on the diffusion of SARS-CoV-2 in mainland China (*27*, *35*, *36*). The model indicates that although the Wuhan travel ban was initially effective at reducing international case importations, the number of imported cases outside mainland China will continue to grow after 2 to 3 weeks. Furthermore, the modeling study shows that additional travel limitations (up to 90% of traffic) have only a modest effect unless paired with public health interventions and behavioral changes that can facilitate a considerable reduction in disease transmissibility (*37*). The model also indicates that, despite the strong restrictions on travel to and from mainland China since 23 January 2020, many individuals exposed to SARS-CoV-2 have been traveling internationally without being detected. Moving forward, we expect that travel restrictions to COVID-19–affected areas will have modest effects and that transmission reduction interventions will provide the greatest benefit for mitigating the epidemic. Our results provide data with potential uses for the definition of optimized containment schemes and mitigation policies, including the local and international dimensions of the COVID-19 epidemic.

## Supplementary Materials

science.sciencemag.org/content/368/6489/395/suppl/DC1 Material and Methods Figs. S1 and S2 Table S1 References (*39*–*110*) MDAR Reproducibility Checklist
